# The effect of nintedanib on lung functions and survival in idiopathic pulmonary fibrosis: real-life analysis of the Czech EMPIRE registry

**DOI:** 10.1186/s12890-023-02450-3

**Published:** 2023-05-03

**Authors:** Marianna Štefániková, Martina Doubková, Petra Ovesná, Martina Šterclová, Ladislav Lacina, Monika Žurková, Martina Plačková, Vladimír Bartoš, Ivana Janíčková, Radka Bittenglová, Jan Anton, Ľubica Sýkorová, Vladimíra Lošťáková, Pavlína Musilová, Hana Šuldová, Radka Mokošová, Jurij Didyk, Lenka Šišáková, Pavlína Lisá, Jaroslav Lněnička, Hana Dařičková, Daniel Doležel, Jana Pšikalová, Richard Tyl, Renata Králová, Martina Koziar Vašáková

**Affiliations:** 1 Department of Pulmonary diseases and tuberculosis, Faculty of Medicine, Masaryk University, University Hospital, Brno, Czech Republic; 2 Institute of Biostatistics and Analyses, Faculty of Medicine, Masaryk University, Brno, Czech Republic; 3 Department of Respiratory Medicine, University Thomayer Hospital, Charles University, Prague, Czech Republic; 4 Department of Pulmonary Medicine and Thoracic Surgery, Hospital Na Bulovce, Prague, Czech Republic; 5 Department of Pulmonary Diseases and Tuberculosis, Faculty of Medicine and Dentistry, Palacky University in Olomouc, University Hospital Olomouc, Olomouc, Czech Republic; 6 Department of Pulmonary Diseases and Tuberculosis, Faculty of Medicine, University of Ostrava, University Hospital Ostrava, Ostrava, Czech Republic; 7 Department of Pulmonary Medicine, Faculty of Medicine in Hradec Kralove at Charles University in Prague, University Hospital Hradec Kralove, Hradec Kralove, Czech Republic; 8 Department of Pulmonary Medicine, University Hospital Plzen, Pilsen, Czech Republic; 9 Department of Pulmonary Medicine, Hospital Jihlava, Jihlava, Czech Republic; 10 Department of Pulmonary Medicine, Hospital Ceske Budejovice, Ceske Budejovice, Czech Republic; 11 Department of Pulmonary Diseases and Tuberculosis, Regional Medical Association, JSC – Masaryk Hospital in Usti nad Labem, Usti nad Labem, Czech Republic; 12 Department of Pulmonary Medicine, Tomas Bata Regional Hospital, Zlin, Czech Republic; 13 Department of Pulmonary Medicine, Faculty of Medicine at Charles University in Prague, University Hospital in Motol, Prague, Czech Republic; 14 Department of Pulmonary Medicine and Allergology, Hospital Kromeriz, Kromeriz, Czech Republic; 15 Department of Pulmonary Medicine, Hospital Novy Jicin, Novy Jicin, Czech Republic; 16 Department of Pulmonary Medicine, Regional Hospital Pardubice, Pardubice, Czech Republic

**Keywords:** Idiopathic pulmonary fibrosis, Nintedanib, Lung function decline, Overall survival

## Abstract

**Introduction:**

The antifibrotic drug nintedanib is used for the treatment of idiopathic pulmonary fibrosis (IPF). We analysed the effect of nintedanib on antifibrotic treatment outcome in real-world cohorts of Czech EMPIRE registry.

**Patients/methods:**

Data of 611 Czech IPF subjects, 430 (70%) treated with nintedanib (NIN group), 181 (30%) with no-antifibrotic treatment (NAF group) were analysed. The influence of nintedanib on overall survival (OS), pulmonary function parameters as forced vital capacity (FVC) and diffusing lung capacity for carbon monoxide (DLCO), as well as GAP score (gender, age, physiology) and and CPI (composite physiological index) were investigated.

**Results:**

During 2 year follow-up we observed that nintedanib treated patients had longer OS, compared to those treated with no-antifibrotic drugs (p < 0.00001). Nintedanib reduces risk of mortality over no-antifibrotic treatment by 55% (p < 0.001). We have observed no significant difference in the rate of FVC and DLCO decline between the NIN and NAF group. Changes within 24 months from baseline in CPI were not significant between the groups (NAF and NIN).

**Conclusion:**

Our real-practice study showed the benefit of nintedanib treatment on survival. There were no significant differences between NIN and NAF groups in changes from baseline in FVC %, DLCO % predicted and CPI.

**Supplementary Information:**

The online version contains supplementary material available at 10.1186/s12890-023-02450-3.

## Introduction

Idiopathic pulmonary fibrosis (IPF) is a specific form of chronic, progressive, fibrosing pneumonia, with unknown etiology associated with the histopathological and/or radiological pattern of usual interstitial pneumonia (UIP) [1]. The disease is characterized by worsening of dyspnea, decline in lung function and is associated with poor prognosis. IPF is a rare disease, which occurs primarily in middle aged to older adults, 67 years (50–82) [2]. Its incidence and mortality seem to be on rise and prevalence increases with aging population [3].

Since 2015 two antifibrotic drugs pirfenidone and nintedanib are recommended in the therapy of IPF. Pirfenidone is an oral antifibrotic drug which reduces disease progression [4, 5] and improves life expectancy [6]. The analysis of the Czech part of the EMPIRE registry observed the 2 years sustained effect of pirfenidone on the decline of lung function and survival in the real-world patients IPF cohort [7]. The effect of pirfenidon on survival in different subgroups of the IPF patients in real world was observed also in the whole cohort of the EMPIRE registry [8].

Nintedanib is a triple kinase inhibitor of platelet-derived growth factor receptor (PDGFR), fibroblast growth factor receptors (FGFR), vascular endothelial growth factor receptor (VEGFR), and Src family kinase, which was approved in treatment of IPF. Studies showed that nintedanib inhibited the proliferation and activation of human fibroblasts and showed antifibrotic and anti-inflammatory activity [9].

In 2014, the efficacy and safety of nintedanib was evaluated in INPULSIS-1 and INPULSIS-2 studies, which were randomized, double-blinded, placebo controlled, parallel-group studies, performed in 24 countries. Both studies showed significantly slower annual rate of decline in FVC in the nintedanib group compared to the placebo group. Moreover in INPULSIS-2, there was a significant difference described in time to first exacerbation over 52 weeks in the nintedanib group compared to placebo [10].

However long term data on disease progression in IPF patients treated with nintedanib is limited and there is a lack of data on the effect of nintedanib in clinical practice. Therefore we decided to conduct a real-life study of Czech EMPIRE registry.

## Patients and methods

Nintedanib has been commercialy available for the patients with IPF in Czechia since September 2016. According to the Czech guidelines, nintedanib is recommended for the mild to moderate stage of IPF patients with measured FVC ≥ 50% and ≤ 90% and predicted DLCO ≥ 30% [11]. This drug is administered twice daily, in the dose 150 mg.

The European MultiPartner IPF registry (EMPIRE, http://empire.registry.cz/index-en.php) is a multinational, observational longitudinal registry designed to describe the characteristics, used treatment and outcomes of IPF patients in 11 Central European countries. The project was launched in 2014, on a basis of the Czech IPF registry, which was founded in 2012 by the Section for ILDs (interstitial lung diseases) of the Czech Pneumological and Phtisiological Society. All subjects are entered consecutively into the registry with their written informed consent and approved by local ethics committees. In our study we used the data of Czech patients entered the EMPIRE registry between January 2016 (availability of nintedanib in the Czech Republic) and April 2022 (the date of analysis).

611 patients from the Czech IPF registry, a national registry within an international multicentre database of IPF patients (EMPIRE) were enrolled to the analysis. All patients were diagnosed according to American Thoracic Society (ATS)/European Respiratory Society (ERS) consensus classification [1] in centers for interstitial lung diseases, followed from September 2016. In this study we analysed patients treated with nintedanib with no history of other antifibrotic treatment (n = 430, 70%) and patients with no antifibrotic treatment (n = 181, 30%). The demographic and clinical data such as sex, age, smoking history, lung function, GAP score, CPI index in enrolled patients were investigated at treatment intitiation and in months 6, 12, 18, and 24 of follow-up (Table 1).

**Table 1:** Baseline characteristics of patients at the time of AF therapy initiation (in the NIN group) or enrollment (in the no-antifibrotic group)

Characteristic	N	Overall, N = 611^1^	NIN, N = 430^1^	NAF, N = 181^1^	p-value^2^
**Men**	611	438 (72%)	324 (75%)	114 (63%)	0.002
**Age at therapy initiation (years)**	611	71 (8)	70 (8)	73 (8)	0.001
**Length of follow-up (months)**	611	17 (6–34)	20 (9–37)	9 (3–26)	< 0.001
**Duration of symptoms (months)**	601	601 / 12 (3; 60)	423 / 12 (3; 60)	178 / 12 (2; 60)	0.007
**Smoking status**	611				0.037
Non-smokers		272 (45%)	202 (47%)	70 (39%)	
Ex-smokers		319 (52%)	218 (51%)	101 (56%)	
Smokers		20 (3.3%)	10 (2.3%)	10 (5.5%)	
**Pharmacological treatment**	611				
Pirfenidone		0 (0%)	0 (0%)	0 (NA%)	
Nintedanib		430 (70%)	430 (100%)	0 (NA%)	
**FVC (L)**	559	2.76 (0.81)	2.75 (0.74)	2.78 (0.93)	0.658
**FVC (%)**	556	81 (18)	78 (14)	88 (23)	< 0.001
**FEV1 (%)**	557	86 (24)	84 (15)	89 (36)	0.075
**DLco (%)**	544	49 (15)	49 (14)	50 (19)	0.375
**GAP index**	551				0.002
I		286 (52%)	185 (49%)	101 (59%)	
II		223 (40%)	171 (45%)	52 (30%)	
III		42 (7.6%)	23 (6.1%)	19 (11%)	
**CPI**	533	46 (37–54)	47 (40–55)	41 (32–50)	< 0.001
**CPI**	533				< 0.001
≤ 41		198 (37%)	115 (31%)	83 (52%)	
> 41		335 (63%)	258 (69%)	77 (48%)	

NIN – patients treated with nintedanib; NAF – patients with no-antifibrotic treatment

^1^n (%); Mean (SD); Median (25–75%)

^2^Pearson’s Chi-squared test; Fisher’s exact test; Welch Two Sample t-test; Wilcoxon rank sum test

### Pulmonary function tests

Spirometry, bodypletysmography, diffusion lung capacity for carbon monoxide were measured according to the ATS/ERS recommendation [12–14]. Results were shown as a percentage of the predicted value, but also as a l/year for FVC and mmol/kPa/min/year for DLCO. In this analysis we included follow-up visits within 24 months from baseline and we focused on the annual rate of change in FVC and DLCO.

### GAP score (gender, age, physiology)

GAP staging was developed in United States and Italy, this staging system stratifies patients with IPF into three categories, using clinical data of gender and age, together with physiological variables (DLCO and FVC) (Table 2). It provides the estimation of mortality in 1-, 2-, 3-year. Patients GAP III are experiencing the worst outcome as shown in Table 3 [15]. However GAP stage does not consider the HRCT (high resolution computed thomography) findings, which is crucial in diagnosing coexisting emphysema. Emphysema can be a counfunding factor, due to increasing % in FVC, which can lead to overestimation of the lung function [16].

**Table 2:** GAP index calculation (15)

	Predictor	Points
G (*gender*)	Female Male	0 1
A (*age*)	≤ 60 61– 65 > 65	0 1 2
P (*physiology*)	FVC > 75 FVC 50–75 FVC < 50 DLco > 55 DLco 36–55 DLco ≤ 35 Cannot perform	0 1 2 0 1 2 3

**Table 3:** Mortality rate according to GAP index (15)

Stage	I	II	III
Points	0–3	4–5	6–8
Mortality 1-y 2-y 3-y1.	5.6 10.9 16.3	16.2 29.9 42.1	39.2 62.1 76.8

In our analysis of the effect of nintedanib treatment, patients where divided into subgroups according to the baseline GAP stage as follows: NIN group: GAP I (N = 185), GAP II (N = 171), GAP III (N = 23). The GAP stage was unavailable for 51 patient from the NIN group. In the NAF group: GAP I (N = 101), GAP II (N = 52), GAP III (N = 19). The adjusted results in each subgroup were adjusted according to the sex, age and baseline level of FVC, DLCO or CPI.

### CPI (composite physiological index)

CPI index is being used to predict the disease progression. CPI is a model which was developed by Wells et al. in a British study. The CPI model is calculated using the percentage of the predicted values of FVC, DLCO and forced exspiratory volume in 1s (FEV_1_) and is associated with the extent of pulmonary fibrosis on high-resolution computed tomography (HRCT). The formula for CPI was as follows: the extent of the disease on HRCT = 91 – (0.65×DLCO percentage of the predicted value [% pred]) − (0.53×FVC % pred) + (0.34×forced expiratory volume [FEV1] % pred) [17]. The advantage of this model is, that it considers the severity of emphysema, which may otherwise lead to overestimation of the lung function [16].

### Statistics

Normally distributed continuous parameters of patients were described by mean and standard deviation (SD) and tested using *t*-test; median and interquartile range (IQR) and Wilcoxon test was applied to other variables. Categorical parameters were described by absolute and relative frequencies. Restricted maximum likelihood estimation based on a random slope and intercept model was used to estimate the adjusted annual rate of decline of lung functions (FVC, DLCO, CPI) from baseline. This model included fixed effects for time, baseline, sex, age as well as treatment-by-time interaction. Random effects for time and intercept were included for each patient. The mean (95% CI) Annual rate of change in lung functions (FVC, DLCO, CPI) from baseline and p-value of statistical significance were reported. Univariate models were followed by the multivariate ones (adjusted for sex, age and baseline level). The significant factors in the univariate models were involved in the multivariate model (see Supplementary Table S1 and S2). Only visits within the first 24 months from the therapy initiation or registry entry was used for the coefficient estimation. At least one post-baseline measurement was required to conduct a random slope and intercept model.

Kaplan-Meier estimate of survival function was used for the visualisation of survival data and it is supplemented by median of survival, number at risk and probability of survival in defined time periods (1 year, 3 years and 5 years after the beginning of the follow-up). Risk factors for overall survival were analysed by Cox proportional hazard model and presented as hazard ratio (HR), both crude and adjusted for baseline values (sex, age and baseline FVC level). All statistical analyses were carried out using R software (R: A language and environment for statistical computing. R Foundation for Statistical Computing, Vienna, Austria. URL https://www.R-project.org/). Level of significance α was set on 0.05. Any p-values presented are considered nominal in nature and no adjustment for multiplicity has been done.

## Results

### Baseline characteristics

Baseline characteristics of IPF patients n = 611; 72% males and 28% females, 430 (70%) treated with nintedanib and 181 (30%) without antifibrotic treatment are presented in the Table 1. The mean age at the time of treatment initiation in the NIN group was 70 years, and 73 years in the NAF group. In the NIN group, mean (SD) FVC at the time of treatment initiation was 78% (14%) of the predicted value, and 88% (23%) in the NAF group (p < 0.001). Mean DLCO was 49% of the predicted value in the NIN group and 50% of the predicted value in the NAF group (p = 0.375). Patients in NIN cohort were divided into subgroups by their baseline GAP index. The mean (SD) value of FVC at the time of treatment initiation was 2.93 l (0.77) in the GAP I group, 2.61 l (0.7) in GAP II group and 2.28 l (0.38) in the GAP III subgroup (p < 0.001). The mean value of DLco at the time of treatment initiation was 57% (12) in the GAP I subgroup, 42% (10) in the GAP II subgroup and 32% (4) in the GAP III subgroup (p < 0.001).

#### Overall survival

**Fig. 1:**
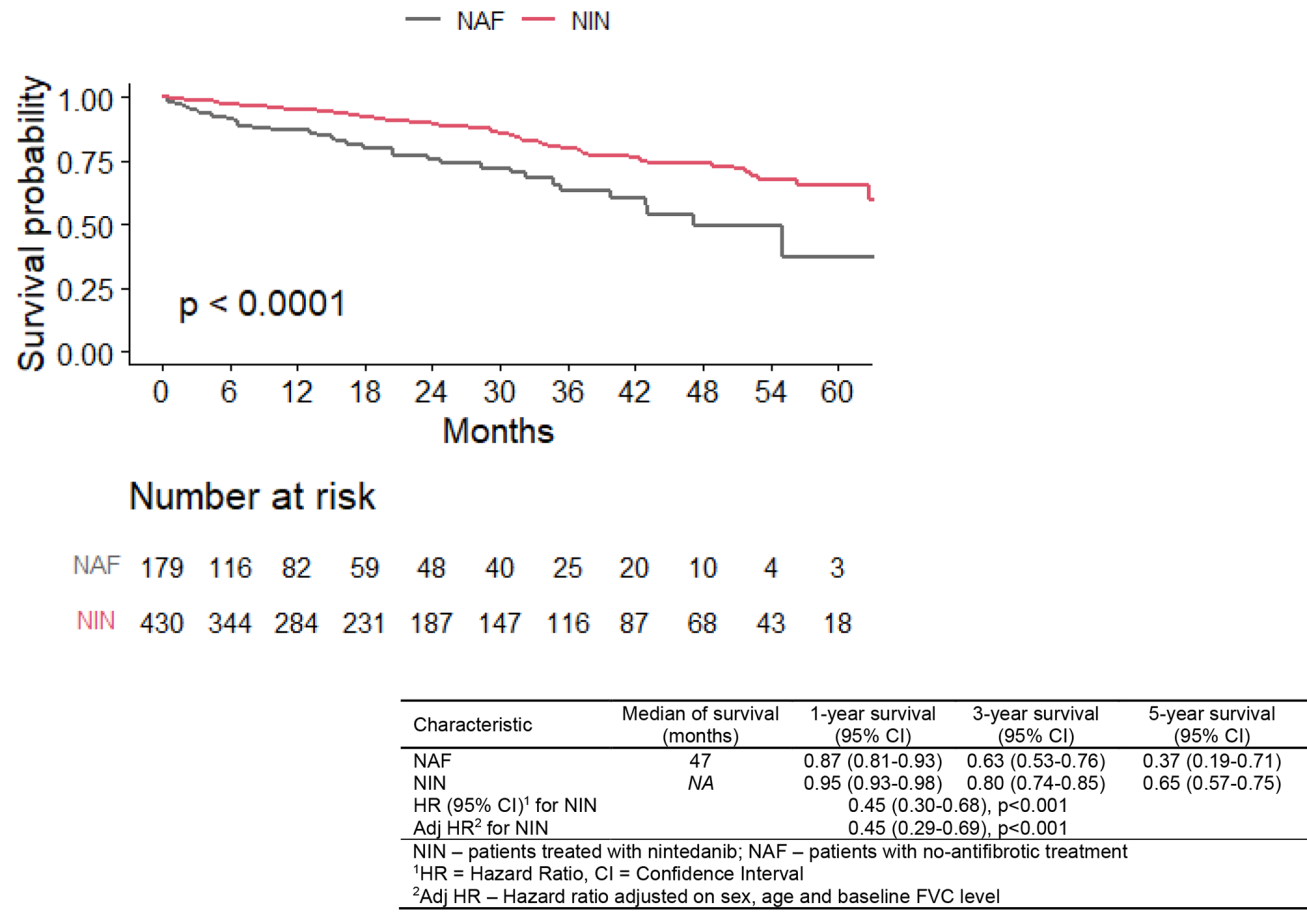
Overall survival of patients with nintedanib (NIN) and with no-antifibrotic (NAF) treatment

We analysed the overall survival (OS) of patients with IPF in NIN and NAF group. The nintedanib treated patients had longer OS, evident at 12, 36, 60 months after treatment initiation, compared to those treated with no-antifibrotic drugs (p < 0.001) as shown in the Fig. 1. The median survival NAF group was 47 months; the median survival was not reached in the NIN group during 5-yr follow-up. Nintedanib reduces risk of mortality over no-antifibrotic treatment by 55% (HR 0.45 (0.3–0.68), p < 0.001).

**Fig. 2:**
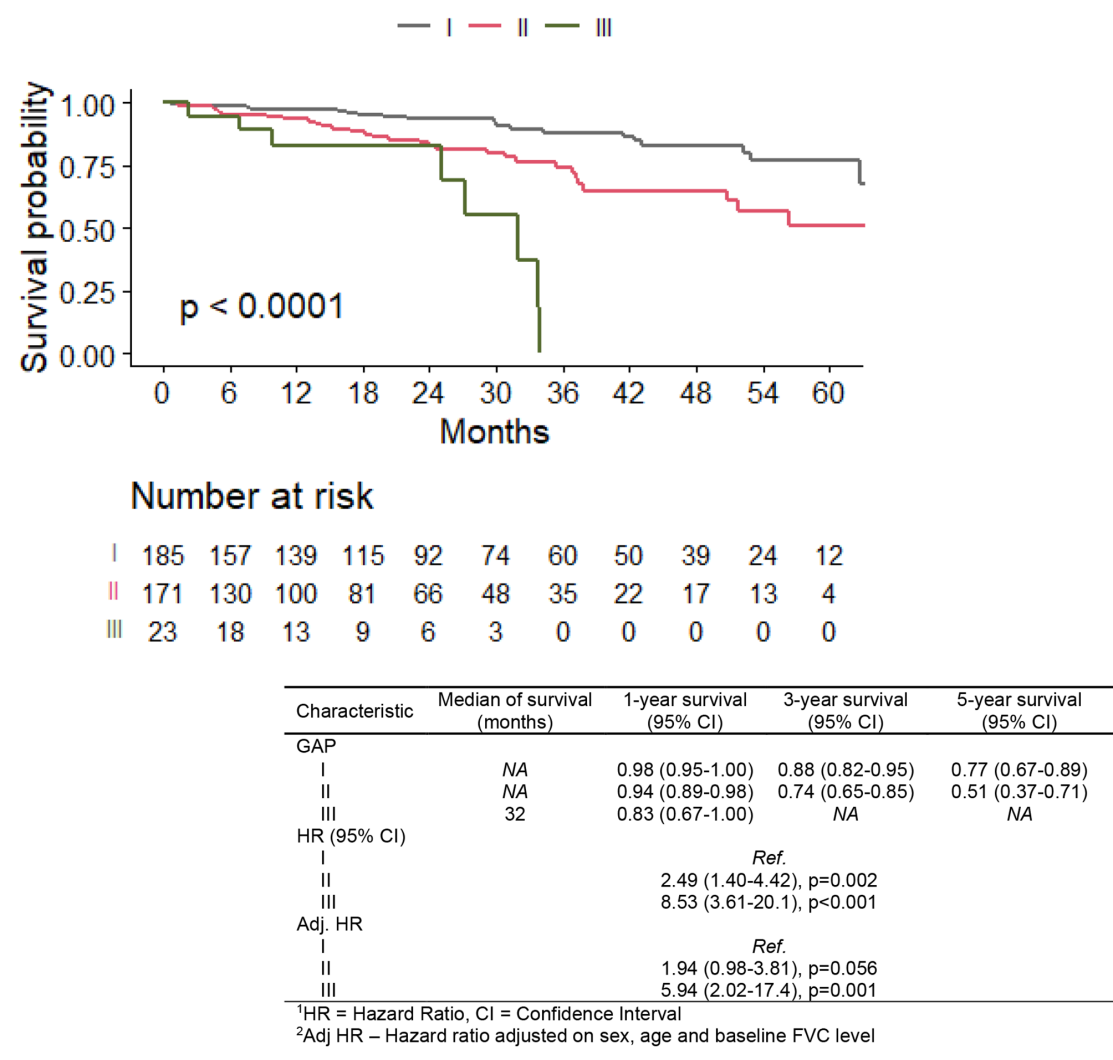
Overall survival of patients with nintedanib treatment with different GAP index at baseline

The OS of the IPF patients in the NIN group, across different GAP stages at the baseline is shown in the Fig. 2., the worst outcome suffer patients in the GAP III subgroup, which is consistent with the predictive function of the GAP stage model.

### Lung functions (FVC, DLCO) and their changes during treatment and follow-up in IPF

**Fig. 3:**
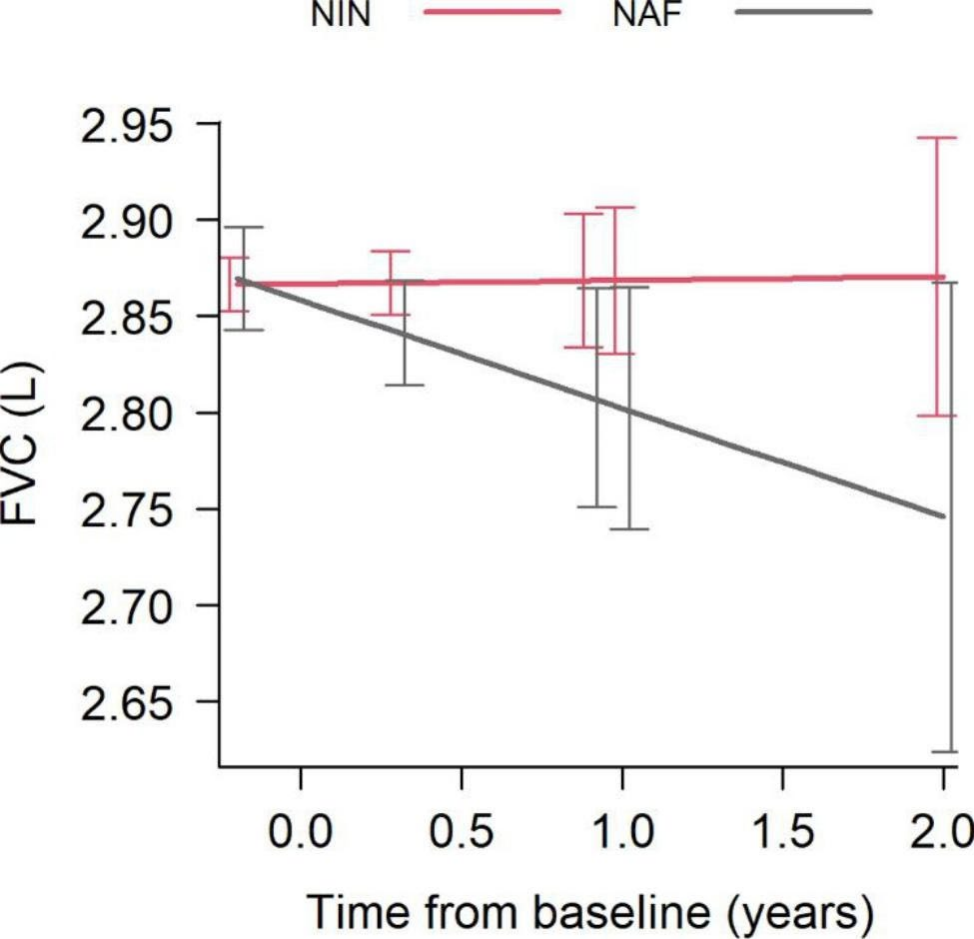
Effect size of the nintedanib (NIN) treatment on FVC (L) compared to no-antifiboric (NAF) group based on the linear model (Table 4, adjusted for sex, age and baseline FVC level)

In this analysis we compared the lung functions during the treatment and the follow-up in NIN and NAF groups as well as in each subgroup according to the baseline GAP stage. The adjusted values in each subgroup were adjusted according to the age, sex and baseline FVC or DLCO level. The mean adjusted annual rate of change in FVC in the NIN group was + 0.002 l/y, p = 0.920, compared to NAF group where mean adjusted annual decline in FVC was − 0.056 l/y, p = 0.071 (Table 4). The difference is not statistically significant, p = 0.108 (Fig. 3). We observed no significant changes in FVC in subgroups of the NIN group (according to their baseline GAP) as shown in the Table 5. The median value of FVC at the time of treatment initiation in the GAP I, II and III group were 3.00, 2.62, 2.30 respectively (p = < 0.001). The annual rate of change in FVC for each subgroup was adjusted according to the age, sex and baseline FVC. The mean adjusted annual decline of FVC in patients with baseline GAP I is -0.005 l/year and − 0.03 l/year, respectively and it did not reach significance between the groups (p = 0.516). In the subgroup with the baseline GAP II, the difference in decline in FVC in NAF group compared to NIN group did not reach statistical significance (+ 0.01 l/year and − 0.092 l/year, p = 0.062 respectively). In the GAP III subgroup, we observed no significant difference in FVC decline betweeen the groups − 0.165 l/y in the NIN group and − 0.144 l/y in the NAF group.

**Table 4:** Linear model for annual change in FVC (L), DLco (mmol/kPa/min) and CPI

*All data are given as*:	Total	NIN	NAF	P^1^
*mean (95% CI), p-value*	**N = 611**	** N = 430**	** N = 181**	
Annual rate of change				
FVC (l/yr)	-0.024 (-0.047; 0), p = 0.050	-0.014 (-0.041; 0.012), p = 0.295	-0.058 (-0.108; -0.008), p = 0.022	p = 0.130
DLco (mmol/kPa/min/yr)	-0.245 (-0.296; -0.195), p < 0.001	-0.27 (-0.327; -0.214), p < 0.001	-0.149 (-0.26; -0.038), p = 0.008	p = 0.056
CPI (per yr)	1.307 (0.584; 2.029), p < 0.001	1.185 (0.352; 2.018), p = 0.006	1.592 (0.124; 3.059), p = 0.034	p = 0.637
Annual rate of change (adjusted^2^)				
FVC (l/yr)	-0.014 (-0.044; 0.017), p = 0.387	0.002 (-0.033; 0.037), p = 0.920	-0.056 (-0.117; 0.005), p = 0.071	p = 0.108
DLco (mmol/kPa/min/yr)	-0.241 (-0.301; -0.18), p < 0.001	-0.266 (-0.335; -0.197), p < 0.001	-0.165 (-0.289; -0.04), p = 0.009	p = 0.163
CPI (per yr)	1.403 (0.451; 2.355), p = 0.004	1.192 (0.091; 2.293), p = 0.035	1.999 (0.102; 3.896), p = 0.039	p = 0.472

*NIN – patients treated with nintedanib; NAF – patients with no-antifibrotic treatment*

^*1*^ *statistical significance of the interaction between time and treatment from the linear mixed-effects model; only follow-up visits within 24 months from baseline were included into the model*

^*2*^ *adjusted for sex, age and baseline level (FVC, DLco or CPI, respectively)*

**Table 5:** Linear model for change in FVC (L) according to GAP index

	NIN		NAF		P^1^	P^2^
	N	Mean (95% CI) (l/yr)	N	Mean (95% CI) (l/yr)		
Annual rate of change in FVC (l/yr)						
GAP I	174	-0.007 (-0.047; 0.033), p = 0.728	101	-0.033 (-0.096; 0.03), p = 0.307	p = 0.499	p = 0.456
GAP II	160	0.004 (-0.04; 0.048), p = 0.853	52	-0.095 (-0.192; 0.002), p = 0.054	p = 0.067	
GAP III	22	-0.166 (-0.296; -0.036), p = 0.012	19	-0.144 (-0.341; 0.054), p = 0.155	p = 0.852	
**Annual rate of change in FVC (l/yr) (adjusted** ^**3**^ **)**						
GAP I	174	-0.005 (-0.045; 0.035), p = 0.799	101	-0.03 (-0.093; 0.033), p = 0.352	p = 0.516	p = 0.437
GAP II	160	0.01 (-0.035; 0.054), p = 0.669	52	-0.092 (-0.189; 0.005), p = 0.064	p = 0.062	
GAP III	22	-0.165 (-0.296; -0.034), p = 0.013	19	-0.144 (-0.343; 0.056), p = 0.158	p = 0.857	

*NIN – patients treated with nintedanib; NAF – patients with no-antifibrotic treatment*

^*1*^ *difference nintedanib vs. no antifibrotics*

^*2*^ *differential effect of nintedanib between GAP index subgroups*

^*3*^ *adjusted for sex, age and FVC (L) at baseline*

The adjusted annual rate of decline in DLCO in the NIN group (-0.266 mmol/kPa/min/yr) compared to NAF group (-0.165 mmol/kPa/min/yr), as also shown in the Fig. 3; Table 4, however this difference was not statistically significant, p = 0.163. When analysing the annual decline in DLCO in NIN patients according to GAP score at baseline, the adjusted annual decline in DLCO in subgroup GAP I-III was similar and it did not reach significance (p = 0.923) (Table 6).

**Table 6:** Comparison of lung functions and their time trends in NIN patients with different GAP index at baseline

*All data are given as*:	GAP I	GAP II	GAP III	P^1^
*mean (95% CI), p-value*	**N = 174**	** N = 160**	** N = 22**	
Annual rate of change				
FVC (l/yr)	-0.007 (-0.05; 0.036), p = 0.750	0.004 (-0.043; 0.052), p = 0.863	-0.173 (-0.314; -0.032), p = 0.016	p = 0.068
DLco (mmol/kPa/min/yr)	-0.261 (-0.345; -0.177), p < 0.001	-0.246 (-0.339; -0.152), p < 0.001	-0.295 (-0.579; -0.011), p = 0.042	p = 0.935
CPI	1.571 (0.374; 2.768), p = 0.011	1.071 (-0.241; 2.383), p = 0.110	-0.825 (-4.917; 3.266), p = 0.693	p = 0.514
Annual rate of change (adjusted^2^)				
FVC (l/yr)	0.011 (-0.039; 0.06), p = 0.666	0.015 (-0.039; 0.069), p = 0.577	-0.183 (-0.345; -0.022), p = 0.026	p = 0.070
DLco (mmol/kPa/min/yr)	-0.267 (-0.37; -0.163), p < 0.001	-0.26 (-0.374; -0.146), p < 0.001	-0.334 (-0.68; 0.012), p = 0.058	p = 0.923
CPI	1.682 (0.195; 3.169), p = 0.028	1.229 (-0.426; 2.884), p = 0.146	-2.817 (-8.075; 2.442), p = 0.294	p = 0.273

^*1*^ *statistical significance of the interaction between time and GAP index from the linear mixed-effects model; only follow-up visits within 24 months from baseline were included into the model*

^*2*^ *adjusted for sex, age and baseline level (FVC, DLCO or CPI, respectively)*

### CPI

There was significant difference between NIN and NAF in CPI at the baseline (at the treatment initiation or the registry entry) (p < 0.001). There was no statistically significant difference in the annual rate of change in CPI between NIN and NAF cohorts (p = 0.637) as well as in the annual rate of change in CPI adjusted for the baseline CPI level (p = 0.472).

## Discussion

The clinical course of IPF is very heterogenous. Due to this fact, the multifactorial predictive models were developed to predict the disease progression. In our study we investigated the effect of nintedanib treatment on lung function and survival as well as the possible impact of predicitve model GAP and CPI and its baseline value on the treatment effect of nintedanib.

According to our data, nintedanib treatment has a positive impact on OS of the IPF patients. The OS of patients treated with nintedanib, across the subgroups by GAP stage at the baseline, is consistent with the predictive values given by GAP. This means that GAP III patients treated with nintedanib suffer the worst outcome.

A decline in FVC over time in patients with IPF is considered an evidence of the disease progression and it is predictive of mortality [1]. We observed no statistical significance in 2yrs sustained effect of nintedanib on decline of FVC. The data also suggest, that there was no difference in the rate of FVC decline between NIN and NAF group. These results are inconsistent with the result of INPULSIS-1 and INPULSIS-2 trials [10]. In the study INPULSIS-1, which confirmed the effect of nintedanib on lung function, the entry criteria were FVC of 50% or more of the predicted value and a DLCO was 30 to 79% of the predicted value (INPULSIS-1). For the study INPULSIS-2, similar inclusion criteria was used (INPULSIS-2). Nintedanib had an effect in patients with more preserved lung function [10].

There was a number of studies, where the effect on nintedanib on FVC decline were analysed across different subgroups, using pooled data from the INPULSIS trials [18–20]. The treatment effect of nintedanib on FVC decline was proved in all above mentioned studies. In our study we observed no significant effect of nintedanib treatment on FVC decline across the subgroups of IPF patients, according to their baseline GAP, unlike the study of Ryerson et al., where the effect of nintedanib on FVC decline was similar in all GAP subgroups, but irrespective of the GAP stage at the baseline [21].

Both factors FVC and DLCO are parts of the GAP predictive model (as described above). Unlike FVC, DLCO is not usually used as a prognostic factor of pulmonary function monitoring in clinical studies with IPF [10]. However Doubková et al. describe DLCO as more accurate predictor of mortality than FVC [2] and Žurková et al. emphasise the importance of the DLCO decline of ≥ 10% as a predictor of mortality in pirfenidon treated patients [7]. However the DLCO values can be distorted by anemia, coexisting emphysema [22] or pulmonary hypertension [23]. There is also a high intra-patient variability in DLCO measurements performed by different technicians, using different equipments [22]. In our study, we observed no significant difference in the annual decline in DLCO in NIN cohort compared to NAF cohort.

Lee et al. compared two multifactorial models which are being used for predicting the IPF progression: GAP stage and CPI index [24]. These models were also used in our analysis. Based on the study of Lee et al., these two models had similar significant predictive values for patients with IPF (p < 0.001) [24]. There was significant difference between NIN and NAF in CPI at the baseline (at the treatment initation) in our study, however the effect of nintedanib on CPI was also not proved. There were two studies by Wells et al. investigating the CPI index and effect of nintedanib, both based on the data from INPULSIS trials. Nintedanib was associated with the marginal effect on CPI index [25] and the lung function decline was slowed down by nintedanib irrespective of baseline CPI index [26].

## Conclusion

In the IPF real-world registry EMPIRE is the overall survival of the patients treated by nintedanib is longer than of those with no antifibrotic treatment. Nintedanib reduces 5yrs risk of mortality over no-antifibrotic treatment by 55% (p < 0.001). We did not observed effect of nintedanib on lung function decline.

## Electronic supplementary material

Below is the link to the electronic supplementary material.

**Supplementary tables**: Table S1. Results of univariate linear models for annual change in FVC (L) and the multivariate model that includes selected significant and independent confounders as factors/covariates. Table S2. Results of univariate Cox proportional-hazards models for overall survival and the multivariate model that includes selected significant and independent confounders as.

## Data Availability

All data generated or analysed during this study are included in this published article.

## Abbreviations

IPF: Idiopathic pulmonary fibrosis
NIN: Nintedanib
NAF: No antifibrotic tratment
OS: Overal survival
FVC: Forced vital capacity
DLco: Diffusing lung capacity for carbon monoxide
GAP: Gender, age, physiology
CPI: Composite physiolgical index
UIP: Usual interstitial pneumonia
PDGFR: Platelet derived growth factor
FGFR: Fibroblast growth factor receptors
VEGFR: Vascular endothelial growth factor
EMPIRE: Eurpean multipartner IPF registry
ATS: American thoracic society
ERS: European respiratory society, HRCT: high resolution computed thomography
FEV 1: Forced exspiratory volume in 1 s
