# Supplementary material for: The effect of nintedanib on lung functions and survival in idiopathic pulmonary fibrosis: real-life analysis of the Czech EMPIRE registry
Source: BMC Pulm Med. 2023 May 3;23:154. doi: 10.1186/s12890-023-02450-3 (PMC10155319; doi:10.1186/s12890-023-02450-3)
Supplement: Supplementary file 1 — Supplementary tables: Table S1. Results of univariate linear models for annual change in FVC (L) and the multivariate model that includes selected significant and independent confounders as factors/covariates. Table S2. Results of univariate Cox proportional-hazards models for overall survival and the multivariate model that includes selected significant and independent confounders as. [file 12890_2023_2450_MOESM1_ESM.docx]

***Supplementary tables***

*Table S1. Results of univariate linear models for annual change in FVC (L) and the multivariate model* *that includes selected significant and independent confounders as factors/covariates*

|  | Univariate models | | | Multivariate model | |
| --- | --- | --- | --- | --- | --- |
| Characteristic |  | Beta (95% CI)^1^ | p-value | Beta (95% CI)^1^ | p-value |
| NIN |  | 0.03 (-0.11; 0.17) | 0.65 | -0.01 (-0.03; 0.02) | 0.52 |
| Men |  | 0.91 (0.79; 1.0) | <0.001 | 0.01 (-0.02; 0.04) | 0.63 |
| Age at therapy initiation (years) |  | -0.02 (-0.03; -0.01) | <0.001 | 0.00 (0.00; 0.00) | 0.62 |
| Length of follow-up (months) |  | 0.01 (0.00; 0.01) | <0.001 | 0.00 (-0.03; 0.04) | 0.92 |
| Duration of symptoms (months) |  | 0.00 (-0.01; 0.00) | 0.032 |  |  |
| GAP index |  |  |  |  |  |
| I |  | — |  |  |  |
| II |  | -0.35 (-0.48; -0.22) | <0.001 |  |  |
| III |  | -0.89 (-1.1; -0.64) | <0.001 |  |  |
| FVC (per 100-ml increase) |  | 0.10 (0.10; 0.10) | <0.001 | 0.10 (0.10; 0.10) | <0.001 |
| FVC (%) |  | 0.02 (0.02; 0.03) | <0.001 |  |  |
| DLCO (mmol/kPa/min) |  | 0.35 (0.31; 0.38) | <0.001 |  |  |
| DLCO (%) |  | 0.02 (0.02; 0.03) | <0.001 |  |  |
| CPI |  | -0.01 (-0.01; -0.01) | <0.001 |  |  |
| Length of follow-up * NIN |  |  |  | 0.06 (-0.01; 0.13) | 0.11 |
| ^1^CI = Confidence Interval | | | | | |

|  | Univariate models | | | Multivariate model | |
| --- | --- | --- | --- | --- | --- |
| Characteristic |  | HR (95% CI)^1^ | p-value | HR (95% CI)^1^ | p-value |
| NIN |  | 0.45 (0.30; 0.68) | <0.001 | 0.45 (0.29; 0.69) | <0.001 |
| Men |  | 0.77 (0.51; 1.17) | 0.22 | 2.13 (1.30; 3.47) | 0.003 |
| Age at therapy initiation (years) |  | 1.03 (1.00; 1.06) | 0.029 | 1.01 (0.98; 1.04) | 0.55 |
| Length of follow-up (months) |  | 0.00 (0.00; 0.00) | <0.001 |  |  |
| Duration of symptoms (months) |  | 1.00 (0.99; 1.01) | 0.91 |  |  |
| GAP index |  |  |  |  |  |
| I |  | — |  |  |  |
| II |  | 2.14 (1.37; 3.33) | <0.001 |  |  |
| III |  | 5.34 (2.79; 10.2) | <0.001 |  |  |
| FVC (L) |  | 0.47 (0.35; 0.63) | <0.001 | 0.36 (0.26; 0.50) | <0.001 |
| FVC (%) |  | 0.97 (0.96; 0.98) | <0.001 |  |  |
| DLCO (mmol/kPa/min) |  | 0.64 (0.54; 0.75) | <0.001 |  |  |
| DLCO (%) |  | 0.96 (0.95; 0.97) | <0.001 |  |  |
| CPI |  | 1.01 (1.00; 1.01) | <0.001 |  |  |
| ^1^HR = Hazard Ratio, CI = Confidence Interval | | | | | |

*Table S2. Results of univariate Cox proportional-hazards models for overall survival and the multivariate model* *that includes selected significant and independent confounders as factors/covariates*
